# USE OF DRUGS IN CHILDREN AGED ZERO TO FIVE YEARS OLD IN TUBARÃO, SANTA CATARINA, BRAZIL

**DOI:** 10.1590/1984-0462/;2018;36;4;00008

**Published:** 2018

**Authors:** Hellen Karoline Maniero, Alexandre Alvares Martins, Angelita Cristine Melo, Leonardo Petrus da Silva Paz, Rosiane de Bona Schraiber, Dayani Galato

**Affiliations:** a Universidade de Brasília, Brasília, DF, Brasil.; b Universidade Federal de São João Del-Rei, Divinópolis, MG, Brasil.; c Universidade do Sul de Santa Catarina, Tubarão, SC, Brasil.

**Keywords:** Drug use, Child, Pharmacoepidemiology, Uso de medicamentos, Criança, Farmacoepidemiologia

## Abstract

**Objective:**

To analyze the context of drug use in children aged zero to five years old.

**Methods:**

Cross-sectional study based on interviews conducted at home with caregivers (parents, uncles or grandparents) of the children enrolled in ten Family Health Strategy units across different geographical points of the city of Tubarão, Santa Catarina, Brazil.

**Results:**

A total of 350 caregivers were interviewed, whose children’s mean age was 2.6 years. Of these, 56.9% had used at least one drug in the 15 days prior to the interview, 31.1% had been exposed to self-medication and 35.7% had used at least one medication obtained by current prescription. The use of medication was associated with the age range up to 24 months, periodic consultation with pediatricians and diagnosis of chronic and acute diseases. Among medicated children, 19.1% inappropriately had been exposed to at least one medication (considering dose, dose interval or period of treatment). Regarding medication storage, 55.2% of interviewees stored them in unsafe places that could be accessed by children and 32.0% in inappropriate places, with exposure to light, heat or humidity. Moreover, 45.2% of the interviewees stored drugs out of their packages, 38.9% without secondary packaging, and 1.6% of drugs had expired date.

**Conclusions:**

Drug use is high among children in this age range, and actions aimed at the safe and rational use of these substances in this population should be encouraged.

## RESUMO

**Objetivo:**

Analisar o perfil de utilização de medicamentos em crianças de zero a cinco anos de idade.

**Métodos:**

Estudo transversal baseado em entrevistas realizadas em domicílio com cuidadores (pais, tios ou avós) das crianças cadastradas em dez unidades de Estratégia Saúde da Família (ESF), distribuídas em diferentes pontos geográficos do município de Tubarão, Santa Catarina.

**Resultados:**

Foram entrevistados 350 cuidadores, cujas crianças sorteadas possuíam, em média, 2,6 anos de idade. Destas, 56,9% utilizaram, pelo menos, um medicamento nos 15 dias anteriores à entrevista, sendo que 31,1% foram expostas à automedicação e 35,7% utilizaram, pelo menos, um medicamento obtido por prescrição atual. O uso de medicamentos foi associado à faixa etária de até 24 meses, consulta periódica com pediatra e diagnóstico de doenças agudas e doenças crônicas. Entre as crianças medicadas, 19,1% foram expostas a pelo menos um medicamento de forma inadequada (considerando dose, intervalo entre doses ou período de tratamento). Quanto ao armazenamento, 55,2% dos medicamentos estavam guardados em lugar inseguro, ou seja, ao acesso das crianças, e 32,0% em locais inadequados, por estarem expostos a luz, calor ou umidade. Ainda, 45,2% estavam sem bula, 38,9% sem embalagem secundária e 1,6% fora do prazo de validade.

**Conclusões:**

As crianças estudadas apresentam uma frequência elevada de uso de medicamentos, devendo ser incentivadas ações que visem ao uso seguro e racional de fármacos nessa população.

## INTRODUCTION

Medicines play a key role in health recovery and maintenance, and should be safe, effective, of suitable quality and affordable for both the patient and the community.^1^ Misuse can pose risks,^2^ which is even more disturbing because the stages of absorption, metabolization, excretion and even effect are different in children’s acceptors compared to what is seen in adults, making this population especially vulnerable to the use of drugs.^3^

In addition, pediatric patients are classified as therapeutic orphans,^3^ that is, for legal, ethical, and economic reasons children are not included in many clinical trials,^2^ ^,^ ^4^ ^,^ ^5^ so the use of drugs on them is done empirically^6^ or even off-label.^7^ Inappropriate use of drugs in this group has been reported in several studies, especially regarding self-medication.^2^ ^,^ ^4^ ^,^ ^8^ ^,^ ^9^ ^,^ ^10^ The high consumption of medicines in childhood can be attributed to the pattern of diseases and clinical manifestations in this age group. In addition to conditions, drug storage in home environment may be another factor inducing medicalization and self-medication,^11^ especially as to systemic-use analgesics, antipyretics and antibiotics.^9^ ^,^ ^11^ It all makes self-medication fairly frequent in this age group,^9^ ^,^ ^12^ and very likely to be kept throughout life, as there is the desire and convenience of managing common childhood complaints without the need for medical consultation.^4^ ^,^ ^11^ ^,^ ^13^

Considering the high prevalence of drug use in children, and self-medication practices by caregivers, one may recognize the importance of promoting the rational use of drugs in this population.^12^ ^,^ ^14^ With that in mind, the objective of this study was to analyze drug use among children aged zero to five years old living in the city of Tubarão, Santa Catarina, Brazil, and factors related.

## METHOD

This is an observational, cross-sectional study based on interviews with caregivers (parents, uncles or grandparents) of children assisted at ten Family Health Strategy (FHS) units in the city of Tubarão, Santa Catarina, Brazil, covering approximately 30% of all children attended by the FHS in the city and geographically distributed to contemplate the cardinal points (north, south, east and west) and the central region, including rural and urban regions. Participants were recruited by convenience sampling. At the time of survey, there were 28 FHS teams in Tubarão, which allowed to cover 90% of its population.

Tubarão is the host city of the Association of Municipalities of Laguna Region (AMUREL) and is a reference for trade and health services and the second most populous city in Southern Santa Catarina, with 97,235 inhabitants according to the 2010 Census.

To determine the minimum sample size, a total of 5,612 children aged up to five years old living in the municipality were considered, according to the 2010 Census. The calculation adopted prevalence of 38.3% as to drug use in this population,^14^ 5% error, and 95% confidence interval (95%CI), which corresponds to a minimum sample of 341 children.

Participants were selected from a list of children in this age group assisted at each FHS unit, being afterwards randomly assigned. The number of patients drawn for interview at each FHS was proportional to the total number of children assisted and, predicting refusals and losses, a second list for random drawn was created for replacements where needed.

The inclusion criteria were: age zero and five years; resident in the city of Tubarão; having been assisted at one of the FHS in the first half of 2012; being previously drawn. The exclusion criteria were: residence not identified; relocation from the city; absence of adult caregivers at the household for interview (after three visits at different and pre-scheduled times).

Upon household visits, the caregivers-parents, uncles or grandparents-were interviewed, as presented previously. In case more than one child in the age group lived in the house, only data from the child selected at the draw would be collected. Up to three attempts to interview the caregiver were made at different days and times before considering “participant loss”. When a caregiver was not present at the household, another suitable time to return and interview would be agreed.

A pilot study was conducted in September 2012, with 10% of the estimated sample in order to evaluate the interview script applicability. Then, the instrument was adjusted for data collection. As there were no substantial changes, these data were also included in the research. All interviews were conducted until December 2012.

The interview was initiated by a presentation of the study objectives, description of how the child was selected, and confirmation of the child’s residence, followed by acceptance by caregivers to participate in the study, with their signing of the informed consent form. After these steps, the interview script would be followed for data collection, the drugs used by the child in the previous 15 days being requested to record information such as name and dosage. Permission was also requested to evaluate the medicine’s storage conditions. In order to collect data, the child’s presence at the household was not necessary. All interviews were conducted by previously trained academics of the Pharmacy course, and data collection was monitored in periodic meetings.

At the time of interview, the script allowed to collect information about the child’s caregivers (relationship to child and remuneration), the family (mother’s and father’s education, income, marital status, other children), and the child (age, sex, gestational age at birth, breastfeeding until sixth month, day care attendance, health insurance coverage, periodicity of consultations with pediatrician, that is, always pre-scheduled from previous consultations; diagnosis of chronic disease), health conditions and use of health services by the child in the 15 days prior to the interview (acute health problems, medical consultations, hospitalization, and use of prescription drugs or self-medication). The health problems mentioned by caregivers as motivators for the use of drugs were categorized as per the International Classification of Diseases (ICD 10) (Collaborating Center of the World Health Organization - WHO for the Classification of Diseases in Portuguese).^15^

The purpose, change in dose or duration of treatment, medical prescription, origin of drugs (mode of acquisition, free access through the Public Health System - SUS, household storage and others) and information related to storage, such as being kept with package insert and secondary packaging and place of storage. Drugs reported to be used by children were organized according to the first level of the Anatomical Therapeutic Chemical (ATC) classification.^16^

The Pediatric & Neonatal Dosage Handbook was used as theoretical reference upon analysis of indication of use adequacy according to age, dose, treatment time, and interval between doses.^17^ Storage suitability and safety was assessed as proposed by Mastroianni et al.,^14^ with storage conditions being considered adequate when no evidence of heat, humidity or clarity was found, considered safe when out of the child’s reach.

The database was input to EpiData version 3.0 (EpiData Association, Odense, Denmark) and analyzed in Epi Info version 6.0 (Centers for Disease Control and Prevention - CDC, Atlanta, USA) and Statistical Package for the Social Sciences (SPSS) version 20.0 (SPSS Inc., Chicago, United States). The numerical variables were presented in measures of central tendency and dispersion, and the nominal variables in absolute numbers and proportions. To assess results, data were distributed in two groups according to drug use in the 15 days prior to interview (considering use by prescription, self-medication or both). Analyses of this outcome (drug use) were compared to other variables using the chi-square test. Subsequently, the variables that resulted p<0.20 in the univariate analysis were used in Forward Stepwise logistic regression. Odds Ratio was estimated by 95%CI and significance level was set at p <0.05.

The research was approved by the Research Ethics Committee of Universidade do Sul de Santa Catarina (CEP Unisul) under number 12.220.4.03.III.

## RESULTS

In order to reach the proper sample size, 361 caregivers of randomly selected children had to be contacted, of whom five were excluded because caregivers were younger than 18 years old, and six because caregivers refused to participate in the study, totaling 350 children. The age range of children was 21 days to 5 years (2.6±1.4 years), of which 52.9% were females. As to drug use, 56.9% (n=199) of the children had been given at least one drug in the past 15 days. Of these, 109 (54.8%) were self-medicated and 125 (62.8%) had valid prescriptions. Important to note that the same child could have been exposed to both modes of drug use. Caregivers’ and family profiles according to use of drugs by children are listed in Tables 1 and 2.

**Table 1:** Profile of caregivers according to drug use by children assisted by Family Health Strategy teams, Tubarão, Santa Catarina, Brazil, 2012.

	Total n (%)	Has used drugs* n (%)	Has not used drugs* n (%)	p-value
Relationship to the child				
Mother	277 (79.1)	168 (60.6)	109 (39.4)	0.018
Other	73 (20.9)	33 (45.2)	40 (54.8)	
Paid job				
Yes	252 (72.2)	142 (56.3)	110 (43.7)	0.560
No	97 (27.8)	58 (59.8)	39 (40.2)	

*in the past 15 days.

**Table 2:** Family profile according to exposure to drug use by children assisted by Family Health Strategy teams, Tubarão, Santa Catarina, Brazil, 2012.

	Total n (%)	Has used drugs* n (%)	Has not used drugs* n (%)	p-value
Mother’s educational level				
Up to 11 years	266 (76.7)	154 (57.9)	112 (42.1)	0.709
12 years and up	81 (23.3)	45 (55.6)	36 (44.4)	
Father’s educational level				
Up to 11 years	245 (82.2)	146 (59.6)	99 (40.4)	0.503
12 years and up	53 (17.8)	29 (57.7)	24 (45.3)	
Marital status				
Partner	300 (85.7)	174 (58.0)	126 (42.0)	0.596
No partner	50 (14.3)	27 (54.0)	23 (46.0)	
Other children				
Yes	204 (58.3)	114 (55.9)	90 (44.1)	0.489
No	146 (41.7)	87 (59.6)	59 (40.4)	
Household income *per capita*				
Up to BRL 550.00	172 (50.1)	100 (58.1)	72 (41.9)	0.876
More than BRL 550.00**	171 (49.9)	98 (57.3)	73 (42.7)	

*in the past 15 days; **Dollar quotation on August 1st 2012: BRL 2,04 equals USD 1,00.

The number of drugs used ranged from one to eight (2.0±1.4) per child, and most of them (76.3%) had been purchased at drugstores. As informed by interviewees, 35.7% of drugs had been prescribed by physicians, while 31.1% had been given to children by the caregiver’s decision. Further data on children’s profile and exposure to drugs are shown in Tables 3 and 4, respectively. The results of the logistic regression analysis are displayed in Table 5.

**Table 3:** Profile of children assisted by Family Health Strategy teams according to drug use, Tubarão, Santa Catarina, Brazil, 2012.

Exposure variables	Total n (%)	Has used drugs* n (%)	Has not used drugs* n (%)	p-value
Age				
Up to 24 months	130 (37.1)	92 (70.8)	38 (29.2)	<0.001
24 months and up	220 (62.9)	109 (49.5)	111 (50.5)	
Gender				
Female	186 (53.1)	112 (60.2)	74 (39.8)	0.262
Male	164 (46.9)	89 (54.3)	75 (45.7)	
Birth gestational age				
Preterm (up to 36 weeks)	57 (16.6)	32 (56.1)	25 (43.9)	0.829
Term (37 or more)	286 (83.4)	165 (57.7)	121 (42.3)	
Breastfed for ≥6 months				
Yes	318 (90.9)	178 (56.0)	140 (44.0)	0.083
No	32 (9.1)	23 (71.9)	9 (28.1)	
Attends day care				
Yes	213 (60.9)	110 (51.6)	103 (48.4)	0.006
No	137 (39.1)	91 (66.4)	46 (33.6)	
Frequent consultations with pediatrician				
Yes	220 (62.9)	142 (64.5)	78 (35.5)	0.002
No	130 (37.1)	59 (45.4)	71 (54.6)	
Diagnosis of chronic diseases				
Yes	104 (37.3)	75 (72.1)	29 (27.9)	<0.001
No	246 (62.7)	126 (51.2)	120 (48.8)	
Usually given drugs by caregiver				
Yes	306 (87.4)	180 (58.8)	126 (41.2)	0.164
No	44 (12.6)	21 (47.7)	23 (52.3)	
Health perception				
Excellent or good	297 (89.2)	156 (52.5)	141 (47.5)	<0.001
Regular or poor	36 (10.8)	30 (83.3)	6 (16.7)	
Child’s health insurance				
Yes	147 (42.0)	93 (63.3)	54 (36.7)	0.060
No	203 (58.0)	108 (53.2)	95 (46.8)	

**Table 4:** Health profile of children assisted by Family Health Strategy teams according to drug use, Tubarão, Santa Catarina, Brazil, 2012.

	Total n (%)	Has used drugs* n (%)	Has not used drugs* n (%)	p-value
Diagnosis of acute diseases*				
Yes	184 (52.6)	160 (87.0)	24 (13.0)	<0.001
No	166 (47.4)	41 (24.7)	125 (75.3)	
Medical consultation*				
Yes	115 (32.9)	99 (86.1)	16 (13.9)	<0.001
No	235 (67.1)	102 (43.4)	133 (56.6)	
Hospitalization*				
Yes	5 (1.4)	4 (80.0)	1 (20.0)	0.304
No	345 (98.6)	197 (57.1)	148 (42.9)	

*in the past 15 days.

**Table 5:** Factors associated with drug use by children assisted at the Family Health Strategy according to the logistic regression model, Tubarão, Santa Catarina, Brazil, 2012.

	*Odds Ratio*	95%CI	p-value
Diagnosis of acute diseases	19.6	10.7-35.8	<0.001
Diagnosis of chronic diseases	2.4	1.2-4.8	0.011
Age (up to 24 months)	2.4	1.2-4.5	0.006
Frequent consultations with pediatrician	2.6	1.4-4.8	0.003

95%CI: 95% confidence interval.

The most used drugs were those acting on the nervous system (24.8%), respiratory system (20.5%), digestive system and metabolism (12.8%), musculoskeletal system (12.2%) and antimicrobials (8.8%). Paracetamol (15.6%), ibuprofen (8.8%), Hedera helix (8.5%), dipyrone (4.8%), amoxicillin (2.8%) and multivitamins (2.5%) were among the most cited drugs. The main purpose of drugs used were: respiratory problems (28.1%), fever (25.6%), gastrointestinal symptoms (10.0%), vitamin and nutrient replacement (9.0%), and conditions related to pain (8.3%).

Bearing in mind the doses administered, the interval between doses and the duration of treatment, 33.7% (n=67) of the children had been inappropriately exposed to at least one drug, with 24 cases of subdose and 20 cases of overdoses.

From the interviews that allowed to evaluate drug storage (n = 124), in 55.2% of cases these were stored in a place the child could have access to. In addition, 32.0% were stored in places considered inadequate, that is, with improper conditions of temperature, humidity and luminosity. Also as to storage, 45.2% of children had been given at least one unlabeled drug, 38.9% at least one drug without secondary packaging (box), and 1.6% at least one drug past expiration date.

## DISCUSSION

Some studies have been conducted to assess the profile of drug use among children in Brazil, but most are related to children who are taken to health services^18^ or are enrolled in educational institutions.^6^ This work, in addition to addressing this population at their households, investigated the use of drugs in a specific age group. Therefore, results are important to the understanding of conditions surrounding it and can help in the definition of actions to promote their rational and safe use. Most of the participating children had been exposed to drugs in the past 15 days by prescription or self-medication.

As for age, children up to 24 months were significantly more medicated than older children. This can be related to the lower capacity of the immune system, which predisposes them to a greater number of diseases, especially infectious, and the greater frequency of medical visits, even when only for monitoring development.^15^ ^17^

No association was found between drug use and gender, as described by Hameen Anttila et al.^18^ However, studies have shown that drug use is significantly higher among males.^19^ ^,^ ^20^

Previous diagnosis of chronic health problems and the habit of consulting a pediatrician periodically were also associated with greater use of drugs. Similar results have been found by other studies^9^ ^,^ ^18^ and suggest that a frequent medical follow-up guarantees the maintenance of treatments already started, as well as generate new drug prescriptions. On the other hand, the high use of drugs may also be related to medicalization in childhood.

The analysis of caregivers’ profile showed that characteristics such as schooling, income and marital status, besides health insurance coverage, did not influence the use of drugs, as described in another study.^21^ Another study found a positive association between these variables, showing that drug use is higher in children with a health plan.^22^ However, cultural and socioeconomic differences between the population of this study and that of international research should be taken into account, as well as between the health systems of each country, for these factors impact on the access to and use of medicines. The free distribution by SUS and the Popular Pharmacy Program broadens the population’s access to medicines and reduces the impact on the family budget.

Similarly, no association was found with the variable gestational age, although morbidities are commonly higher among children born prematurely.^23^ The variables breastfeeding and number of children were also not associated with drug use, although previous studies have shown the opposite.^24^ In addition, a study reported that children from families with other children made fewer visits to pediatricians and were less likely to use prescription drugs.^21^

Self-medication often resulted from the re-use of old prescriptions and drug-related unlabeled drugs, which does not make it safer in this population.^25^ According to Beckhauser et al., this is usually considered more practical, with re-use being more frequent in younger children.^9^

In this study, 33.7% of children had been exposed to at least one drug inappropriately. This may be related to the choice of ineffective treatment, drugs with the same active principles, inadequate dose, among others.26 Therefore, patients should be advised of possible problems resulting from this practice.

Analgesic/antipyretic drugs were predominantly used among interviewees, as reported by Pereira et al.^27^ Indications for fever management are usually related to the discomfort experienced by the child. However, in the caregivers’ view, fever is always considered a serious problem that must be tackled.^6^ Supporting this data, paracetamol was the mostly cited drug in this and other studies,^20^ ^,^ ^21^ which highlights the risk of adverse events related to their use.^25^ ^,^ ^28^ Respiratory problems were the most commonly mentioned in the research and may be associated with acute diseases (87%), which supports the consumption of Hedera helix.

The results show the access to drugs by the population, however problems were observed as to use suitability. Inadequate use was identified in 33.7% of the sample, which can generate expenses in health services by preventable events, with increase in the number of hospital visits, hospitalizations and deaths.^29^ Another study has shown that about 15% of hospital admissions in Brazil are related to drug adverse effects.^30^ This leads to the possibility of intoxications,^26^ therapeutic failures or even treatment abandonment due to lack of knowledge by caregivers, divergences in the orientation process or even the practice of self-medication. It is also important to highlight the potential of FHS, which can work out this problem and reduce the consequences of irrational drug use as there is a continuous interaction between different health professionals and diversity of knowledge and skills.

Regarding storage, a significant number of drugs is stored in unsafe, inadequate places. This finding is comparable to those reported by Tourinho et al.^11^ and Mastroianni et al.^14^, and suggests the need for interventions aiming to promote the rational drug use in this population, especially by FHS professionals and including health community agents.

Drug use profile should be considered an indicator of health problems in a given population, besides being able to point the deficiencies and advantages of the health system, drug regulation, medical education, cultural habits, pharmaceutical market composition, and other factors.^2^

The study design and selection of FHS units by convenience can be considered limitations. It should be noted that such limitations may impair the external validity of the research, that is, the possibility of extrapolating findings.

This study is relevant to demonstrate the need for public policies aimed at actions in Pediatrics. Child care begins with the effective orientation of caregivers, which can be achieved in a health care system that is committed to efficiency and quality.

## References

[1] Organización Mundial de la Salud Perspectivas políticas sobre medicamentos de la OMS - Promoción del uso
racional de medicamentos: componentes centrales 2002.

[2] Cruz MJB, Dourado LFN, Bodevan EC, Andrade RA, Santos DF (2014). Medication use among children 0-14 years old: population baseline
study. J Ped.

[3] Meiners MMMA, Bergstein-Mendes G (2001). Prescrição de medicamentos para crianças hospitalizadas: como
avaliar a qualidade?. Rev Assoc Med Bras.

[4] Telles PCP, Pereira AC (2013). Self-medication in children from zero to five years: farmacos
managed, knowledge, statement and background. Esc Anna Nery Rev Enferm.

[5] Moraes CG, Mengue SS, Tavares NUL, Dal Pizzol TS (2013). Drug use among children between zero and six years old: a
population baseline study in the south of Brazil. Ciênc Saúde Coletiva.

[6] Carvalho DC, Trevisol FS, Menegali BT, Trevisol DJ (2008). Drug utilization among children aged zero to six enrolled in day
care centers of Tubarão, Santa Catarina, Brazil. Rev Paul Pediatr.

[7] Costa PQ, Rey LC, Coelho HLL (2009). Lack of drug preparations for use in children in
Brazil. J Pediatr (Rio J).

[8] Pereira FSVT, Bucaretchi F, Stephan C, Cordeiro R (2007). Self-medication in children and adolescents. J Pediatr (Rio J).

[9] Beckhauser GC, Souza JM, Valgas C, Piovezan AP, Galato D (2010). Medication use in Pediatrics: the practice of self-medication in
children by their parents Rev Paul. Pediatr.

[10] Du Y, Knopf H (2009). Self-medication among children and adolescents in Germany:
results of the National Health Survey for Children and Adolescents
(KiGGS). Br J Clin Pharmacol.

[11] Tourinho FSV, Bucaretchi F, Stephan C, Cordeiro R (2008). Home medicine chests and their relationship with self-medication
in children and adolescents. J Pediatr (Rio J).

[12] Oliveira EA, Bertoldi AD, Domingues MR, Santos IS, Barros AJD (2010). Medicine use from birth to age two years: the 2004 Pelotas
(Brazil) Birth Cohort Study. Rev Saúde Pública.

[13] Santos DB, Barreto ML, Coelho HLL (2009). Drug use and associated factors in children living in poor
areas. Rev Saúde Pública.

[14] Mastroianni PC, Lucchetta RC, Sarra JR, Galduróz JCF (2011). Estoque doméstico e uso de medicamentos em uma população
cadastrada na estratégia saúde da família no Brasil. Rev Panam Salud Pública.

[15] Thrane N, Sorensen HT (1999). A one-year population-based study of drug prescriptions for
Danish children. Acta Paediatr.

[16] Pedraza DF, Queiroz D, Sales MC (2014). Infectious diseases among Brazilian preschool children attending
daycare centers. Ciênc Saúde Coletiva.

[17] Overturf GD (1994). Editorial Response: Endemic giardiasis in the United States--role
of the daycare center. Clin Infect Dis.

[18] Hameen-Anttila K, Lindell-Osuagwu L, Sepponen K, Vainio K, Halonen P, Ahonen R (2010). Factors associated with medicine use among children aged under 12
years--a population survey in Finland. Pharmacoepidemiol Drug Saf.

[19] Cazzato T, Pandolfini C, Campi R, Bonati M (2001). Drug prescribing in out-patient children in Southern
Italy. Eur J Clin Pharmacol.

[20] Straand J, Rokstad K, Heggedal U (1998). Drug prescribing for children in general practice. A report from
the More & Romsdal Prescription Study. Acta Paediatr.

[21] Chen AY, Escarce JJ (2006). Effects of family structure on children's use of ambulatory
visits and prescription medications. Health Serv Res.

[22] Pizzol TSD, Tavares NUL, Bertoldi AD, Farias MR, Arrais PSD, Ramos LR (2016). Use of medicines and other products for therapeutic purposes
among children in Brazil. Rev Saúde Pública.

[23] Ramos HAC, Cuman RKN (2009). Risk factors for prematurity: document search. Esc Anna Nery Rev Enferm.

[24] Oddy W, Sly P, de Klerk NH, Landau L, Kendall G, Holt P (2003). Breast feeding and respiratory morbidity in infancy: a birth
cohort study. Arch Dis Child.

[25] Trajanovska M, Manias E, Cranswick N, Johnston L (2010). Use of over-the-counter medicines for young children in
Australia. J Paediatr Child Health.

[26] Montastruc JL, Bondon-Guitton E, Abadie D, Lacroix I, Berreni A, Pugnet G (2016). Pharmacovigilance, risks and adverse effects of
self-medication. Therapie.

[27] Pereira GL, Tavares NU, Mengue SS, Pizzol Tda S (2013). Therapeutic procedures and use of alternating antipyretic drugs
for fever management in children. J Pediatr (Rio J).

[28] Barzaga Arencibia Z, Choonara I (2012). Balancing the risks and benefits of the use of over-the-counter
pain medications in children. Drug safety.

[29] Arrais PSD, Coelho HLL, Batista MCDS, Carvalho ML, Righi RE, Arnau JM (1997). Aspects of self-medication in Brazil. Rev Saúde Pública.

[30] Mastroianni PC, Varallo FR, Barg MS, Noto AR, Galduróz JCF (2009). Contribuição do uso de medicamentos para a admissão
hospitalar. Braz J Pharm Sci.

